# Dopamine system as a mediator between psychological symptoms and relapse tendency: a cross-sectional study in women undergoing compulsory rehabilitation in Zhengzhou, China

**DOI:** 10.3389/fpubh.2026.1816411

**Published:** 2026-04-28

**Authors:** Chunxia Jin, Yuxia Chen, Kai Xu, Bensheng Guo, Hai Li, Zijian Zhao

**Affiliations:** 1 College of Health Promotion, Gdansk University of Physical Education and Sport, Gdansk, Poland; 2 Huanghe Science and Technology University, Zhengzhou, China; 3 Henan Sports Science and Technology Center (Henan Anti-Doping Center), Zhengzhou, China; 4 Faculty of Health Sciences and Sports, Macao Polytechnic University, Macau, China; 5 College of Sport, Neijiang Normal University, Neijiang, China; 6 School of Kinesiology and Physical Education, Zhengzhou University, Zhengzhou, China

**Keywords:** dopamine, drug dependence, mediation effect, psychological symptoms, relapse propensity

## Abstract

To examine whether psychological symptoms influence relapse propensity among individuals with drug dependence through the dopamine (DA) system, after controlling for confounders such as age. Ninety Women Individuals in Compulsory Drug Rehabilitation, aged 18–60 years, were enrolled. Key measures included psychological status [assessed via the Montreal Cognitive Assessment (MoCA), Self-rating Depression Scale (SDS), Symptom Checklist-90 (SCL-90), and Pittsburgh Sleep Quality Index (PSQI)]; physiological indicators [blood concentrations of serotonin (5-HT), interleukin-6 (IL-6), gamma-aminobutyric acid (GABA), and dopamine (DA)]; and socio-demographic and drug-use characteristics (age, education, marital status, years of drug use, etc.). Relapse propensity was quantified using a Relapse Propensity Scale score. Data analysis included Pearson correlation, stepwise multiple linear regression, and mediation analysis using the bootstrap method (PROCESS Model 4). Bivariate analysis revealed that relapse propensity was significantly correlated with age (*r* = 0.322, *p* < 0.01), SCL-90 GSI (*r* = 0.261, *p* < 0.05), and DA concentration (*r* = 0.341, *p* < 0.01). Stepwise regression identified DA concentration (*β* = 0.321, *p* = 0.001) and age (*β* = 0.301, *p* = 0.002) as the only significant independent predictors, jointly accounting for 18.8% of the variance in relapse propensity (adjusted R^2^ = 0.188). Crucially, mediation analysis confirmed a significant indirect effect of SCL-90 on relapse propensity through DA (indirect effect = 0.018, 95% CI [0.005, 0.035]). The direct effect was non-significant (*p* = 0.170), indicating full mediation. The mediated effect accounted for 35.6% of the total effect. This study indicates that psychological symptoms indirectly increase relapse propensity in Compulsory Drug Rehabilitation by elevating DA concentration. DA fully mediates the relationship between psychological symptoms and relapse propensity. Age independently predicts relapse propensity but does not act through the DA system. These findings support an integrated “psychological–neurobiological” intervention approach. In clinical practice, relapse prevention strategies should combine psychological symptom management with DA system modulation, alongside age-tailored interventions.

## 1 Introduction

Relapse following substance use treatment represents one of the most persistent challenges in global public health, with annual relapse rates remaining as high as 40–60% despite systematic interventions (1). This high rate of relapse inflicts profound damage on individual health and social functioning while imposing a sustained burden on public health systems (2–5). A critical step toward developing more effective interventions is to elucidate the complex mechanisms underlying relapse behavior.

Relapse is influenced by a confluence of biological, psychological, and social factors. At the psychological level, symptoms of depression, anxiety, and general distress are highly prevalent among individuals with substance dependence. These symptoms are thought to directly potentiate drug craving and impair behavioral control, thereby elevating relapse risk (6–8). At the neurobiological level, the dopamine (DA) system is central to reward processing, motivation, and impulse control. Dysregulation of this system is strongly implicated in the maintenance of addictive behaviors and vulnerability to relapse (9–12). Chronic substance use induces enduring neuroadaptations within DA pathways, which help perpetuate the addiction cycle (13–15).

Although research has separately established the roles of psychological distress and DA dysregulation in relapse (16), the integrative pathway through which psychological factors interact with this core neurochemical system to influence behavior remains poorly understood. Theoretical models posit that psychological symptoms, particularly stress and negative affect, may indirectly modulate DA function through the activation of neuroendocrine (e.g., HPA axis) and inflammatory processes (17–19). This suggests a plausible neurobiological mediation model: psychological distress could increase relapse propensity by altering DA system activity (20). However, direct empirical tests of this “psychological-neurochemical” pathway are scarce, as most studies examine these domains in isolation. Furthermore, age—a key demographic variable linked to duration, neural adaptation, and psychosocial resources (21, 22)—is often overlooked as a potential confounder or moderator in this relationship.

Despite extensive research documenting the separate roles of psychological distress and dopamine dysregulation in addiction relapse, a critical gap remains in the literature: no prior study has empirically tested whether dopamine serves as a neurobiological mediator linking psychological symptoms to relapse propensity in individuals with substance use disorders. The present study aims to address this gap by proposing and empirically testing an integrated “psychological-neurobiological” model.

In this study, “psychological symptoms” are operationalized as general psychological distress, measured by the Global Severity Index (GSI) of the Symptom Checklist-90 (SCL-90), which encompasses a broad spectrum of psychopathological symptoms including anxiety, depression, and hostility. We propose the following directional hypotheses: (H1) General psychological symptom (SCL-90 GSI) burden is positively correlated with both peripheral DA concentration and self-reported relapse propensity; (H2) DA concentration mediates the relationship between psychological symptoms and relapse propensity, such that psychological symptoms indirectly increase relapse risk by elevating DA levels; and (H3) Age contributes independently to relapse propensity, and this effect is not mediated through the DA system. The corresponding null hypothesis is that dopamine does not mediate the relationship between psychological symptoms and relapse propensity.

Accordingly, the specific objectives of this study are threefold: (1) to examine the bivariate correlations among psychological symptoms (SCL-90 GSI), DA concentration, and relapse propensity; (2) to identify the independent predictors of relapse propensity using multivariate analysis; and (3) to formally test the mediating role of DA concentration in the pathway from psychological symptoms to relapse propensity, while statistically controlling for the influence of age.

By directly investigating this integrative mechanism, the present research not only advances the theoretical understanding of relapse by bridging psychological and neurochemical perspectives but also provides critical empirical evidence to inform the development of targeted, multi-modal intervention strategies that concurrently address psychological distress and its neurobiological sequelae.

The innovative value of this research lies in its integrative empirical test of a theoretically plausible yet rarely examined neurobiological mediation model in a clinical sample. By moving beyond simple correlations to probe the directional and mechanistic interplay between mind and molecule, this work seeks to provide a more nuanced explanation for relapse vulnerability. Confirmation of this pathway would significantly shift clinical perspectives, highlighting the dopaminergic system as a key treatment target not only for reward processing deficits but also for the relapse risk conferred by psychological comorbidities, thereby informing the development of more effective, integrated intervention strategies.

## 2 Methods

### 2.1 Trial design and sample size

This study employed a cross-sectional design. All data were collected during a single, comprehensive assessment session conducted in May 2024 at the Women’s Compulsory Drug Rehabilitation Center in Henan Province, China. A consecutive sampling method was used, whereby all eligible women admitted during the study period were invited to participate.

No *a priori* sample size calculation was performed due to the exploratory nature of the study and the fixed population available during the data collection period. To evaluate the adequacy of the obtained sample (*N* = 90) for detecting the primary mediation effect, a post-hoc statistical power analysis was conducted using G*Power 3.1.9.7 (52). For the mediation model (test of the indirect effect), with a conservatively estimated effect size of f^2^ = 0.09 (based on the observed R^2^ increment in the full mediation model), *α* = 0.05, and three predictors in the full model, the achieved power was 0.81, exceeding the conventional threshold of 0.80. This indicates that the sample size was adequate to detect the hypothesized mediation effect with sufficient statistical power.

The primary objective was to examine the interrelationships between psychological symptom severity, peripheral neurochemical concentrations, and self-reported propensity to relapse among a cohort of women undergoing compulsory rehabilitation (23) for substance use disorders.

### 2.2 Participants

Participants were women meeting the following inclusion criteria: (1) aged between 18 and 60 years; (2) a confirmed diagnosis of Substance Use Disorder (Opioid or Stimulant type) according to the Diagnostic and Statistical Manual of Mental Disorders, Fifth Edition (DSM-5) (24); (3) being in a clinically stable period of abstinence, defined as having discontinued all illicit substance use for a minimum of 1 month prior to assessment (25); (4) sufficient literacy and cognitive capacity to provide informed consent and complete self-report questionnaires. Exclusion criteria were: (1) a comorbid diagnosis of a major psychotic or neurological disorder (e.g., schizophrenia); (2) the presence of a severe, unstable medical condition or acute infectious disease; (3) reported use of any psychotropic, anti-inflammatory, or corticosteroid medication within the preceding week, due to their potential confounding effects on neuroendocrine and immune markers. A consecutive sampling method was used, meaning all eligible women admitted to the center during the study period (May 2024) were invited to participate. No randomization was applied, which is appropriate for this cross-sectional design. After screening, a final sample of 90 eligible participants was enrolled. The study protocol received full ethical approval from the Institutional Review Board of the Women’s Compulsory Drug Rehabilitation Center in Henan Province (Approval No. 202401). The study’s purpose, procedures, potential risks, and benefits were explained in detail to all potential participants. Written informed consent was obtained from every individual prior to their inclusion in the study.

### 2.3 Measures and data collection

Data collection was standardized and performed by trained research staff in a private setting within the facility. The assessment battery included:

#### 2.3.1 Demographic and clinical variables

A researcher-administered interview collected data on age, years of education, marital status, primary substance of abuse, and total duration of substance use.

#### 2.3.2 Psychological and behavioral measures: protocol standardization

- **General Psychological Distress:** The Chinese version of the Symptom Checklist-90 (SCL-90) (26, 27) was used to assess a broad spectrum of psychopathological symptoms over the past week. The Global Severity Index (GSI), calculated as the mean score of all 90 items, served as the primary continuous measure of overall psychological symptom burden. The scale demonstrated excellent internal consistency in the present sample (Cronbach’s *α* = 0.89).
- **Depressive Symptoms:** The Self-Rating Depression Scale (SDS) was employed to provide a specific measure of depressive symptom severity (28) (Cronbach’s *α* = 0.84).
- **Sleep Quality:** Sleep disturbance over the preceding month was evaluated using the Pittsburgh Sleep Quality Index (PSQI). A global PSQI score >5 is typically indicative of poor sleep quality (26, 27). The PSQI showed acceptable internal consistency (Cronbach’s α = 0.79).
- **Relapse Propensity:** The Relapse Tendency Questionnaire is a 18-item self-report tool adapted from Tiffany’s cognitive model and validated in Chinese populations (26, 27). This instrument captures dimensions of craving intensity, perceived risk in high-risk situations, and behavioral intention to use substances. The total score was used for analysis, with higher scores indicating a greater propensity for relapse. The scale showed good reliability (Cronbach’s α = 0.90).

#### 2.3.3 Biological sample collection and neurochemical assay

Fasting venous blood samples (approximately 10 mL) were drawn from each participant between 7:00 and 9:00 a.m. Samples were immediately centrifuged, and the separated plasma was aliquoted and stored at −80 °C until batch analysis. Concentrations of key neurochemical analytes were quantified using commercially available, high-sensitivity enzyme-linked immunosorbent assay (ELISA) kits according to the manufacturers’ protocols. All assays were performed in duplicate, and the mean value was used for statistical analysis. The analyzed markers included:

- **Dopamine (DA):** The primary neurochemical variable of interest (measured in nmol/L). The intra-assay and inter-assay coefficients of variation (CV) were < 8% and < 12%, respectively, confirming assay precision.
- **Serotonin (5-HT):** Measured in ng/mL.
- **Interleukin-6 (IL-6):** A pro-inflammatory cytokine, measured in ng/L.
- **Gamma-Aminobutyric Acid (GABA):** The primary inhibitory neurotransmitter, measured in mmol/L.

### 2.4 Statistical analysis

All statistical analyses were performed using IBM SPSS Statistics (Version 27.0, Armonk, NY, United States) and the PROCESS macro (Version 4.1) for mediation analysis. A two-tailed probability value of *p* < 0.05 was considered statistically significant.

#### 2.4.1 Data screening and descriptive statistics

The dataset was initially screened for accuracy and completeness. There were no missing data for any of the key variables (demographics, SCL-90, DA, age, relapse propensity) among the 90 participants, allowing for a complete-case analysis. Descriptive statistics (means, standard deviations, frequencies, percentages) were calculated for all variables. The normality of distribution for continuous variables was examined using the Kolmogorov–Smirnov test and visual inspection of Q-Q plots. Given the sample size (*N* = 90) and the approximate normality of the core variables, parametric statistical methods were deemed appropriate.

#### 2.4.2 Bivariate correlation analysis

Zero-order Pearson correlation coefficients were computed to examine the linear associations among all demographic, psychological, neurochemical, and outcome variables. This step identified variables significantly associated with relapse propensity for inclusion in subsequent multivariate models.

#### 2.4.3 Multivariate regression analysis (predictor identification)

To identify the most parsimonious set of independent predictors of relapse propensity, a forward stepwise multiple linear regression was conducted. This approach combines forward selection with backward elimination. All variables demonstrating a significant bivariate correlation with relapse propensity (*p* < 0.10) or strong theoretical relevance were entered as potential predictors. The stepwise procedure used criterion probabilities of *p* < 0.05 for entry and *p* > 0.10 for removal. Multicollinearity among predictors in the final model was assessed using Variance Inflation Factors (VIF), with a VIF > 10 indicating a problematic level of collinearity.

#### 2.4.4 Hypothesis testing: mediation analysis

To formally test the central hypothesis that DA concentration mediates the relationship between psychological distress and relapse propensity, a simple mediation analysis was conducted using Hayes’ PROCESS Model 4. The independent variable was the SCL-90 GSI score (X), the mediator was DA concentration (M), and the dependent variable was relapse propensity score (Y). Given its significant association with the outcome, age was included as a covariate in the model. The significance of the indirect effect (path a * b) was tested using a bias-corrected bootstrapping method with 5,000 resamples. A 95% confidence interval (CI) for the indirect effect that did not include zero was considered evidence of significant mediation. The analysis provided estimates for the total effect (c), direct effect (c’), and indirect effect (a*b) of X on Y.

#### 2.4.5 Model diagnostics

For the final regression and mediation models, assumptions of linearity, homoscedasticity, and independence of residuals were evaluated by examining scatterplots of standardized residuals against standardized predicted values. The normality of residuals was assessed using normal probability plots (P–P plots). The Durbin-Watson statistic was consulted to check for autocorrelation of residuals in the regression model.

## 3 Results

### 3.1 Descriptive statistics

A total of 90 participants were included in the final analysis. The demographic, psychological, and physiological characteristics of the sample are summarized in Table 1. The mean age of participants was 44.73 years (SD = 10.95). The majority of participants had a high school education or below (95.6%). The mean score for psychological symptoms (SCL-90) was 152.12 (SD = 49.47), indicating a moderate level of distress. The mean dopamine (DA) concentration was 1.70 nmol/L (SD = 0.86), and the mean relapse propensity score was 38.54 (SD = 11.68).

**Table 1:** Demographic, clinical, psychological, and neurobiochemical characteristics of the study participants (*N* = 90).

Variable	Mean ± SD or n (%)
Descriptive statistics	
Age (years)	44.73 ± 10.95
Education	
High school or below	86 (95.6%)
College / University	4 (4.4%)
Marital status	
Married	27 (30.0%)
Single	31 (34.4%)
Divorced	25 (27.8%)
Widowed	7 (7.8%)
Substance type	
Stimulants (primarily methamphetamine)	23 (25.6%)
Methamphetamine (crystal meth)	12 (13.3%)
Ketamine	11 (12.2%)
Opioids (primarily heroin)	71 (78.9%)
Cannabis	3 (3.3%)
Other	2 (2.2%)
Polysubstance Use (two or more types)	9 (10.0%)
Years of drugs use	
≤5	16 (17.8%)
5–10	11 (12.2%)
10–20	42 (46.7%)
>20	21 (23.3%)
Psychological variables	
MOCA score	23.40 ± 5.24
SDS score	41.20 ± 8.75
SCL-90 score	152.12 ± 49.47
PSQI sleep score	7.09 ± 3.61
Physiological variables	
5-HT (ng/ml)	89.02 ± 49.66
IL-6 (ng/l)	1.31 ± 0.56
GABA (mmol/l)	0.03 ± 0.15
DA (nmol/l)	1.70 ± 0.86
Outcome variable	
Relapse propensity score	38.54 ± 11.68

Continuous variables are presented as Mean ± Standard Deviation. Categorical variables are presented as n (%). MoCA, Montreal Cognitive Assessment; SCL-90, Symptom Checklist-90; GABA, Gamma-aminobutyric acid; 5-HT, 5-Hydroxytryptamine; DA, Dopamine.

### 3.2 Bivariate correlational analyses

Pearson correlation coefficients among all study variables are presented in Table 2. Relapse propensity showed significant positive correlations with Age (*r* = 0.322, *p* < 0.01), psychological symptoms as measured by the SCL-90 (*r* = 0.261, *p* < 0.05), sleep quality problems measured by the PSQI (*r* = 0.277, *p* < 0.01), and DA concentration (*r* = 0.341, *p* < 0.01). No other variables, including other neurochemical markers (5-HT, IL-6, GABA), cognitive function (MOCA), or depression (SDS), were significantly correlated with relapse propensity at the bivariate level.

**Table 2:** Correlations between study variables and relapse tendency (*N* = 90).

Variable	r	*p*
Age (years)	0.322	0.002**
Education Level	−0.047	0.658
Marital Status	−0.03	0.777
Duration of Drug Use (years)	0.009	0.936
MoCA Score	0.039	0.709
SDS Score	0.149	0.162
SCL-90 GSI Score	0.261	0.013*
PSQI Score	0.277	0.008**
5-HT Concentration (ng/ml)	0.165	0.12
IL-6 Concentration (ng/l)	0.07	0.511
GABA Concentration (mmol/l)	−0.006	0.958
DA Concentration (mmol/l)	0.344	0.001**

MoCA, Montreal Cognitive Assessment; SDS, Self-Rating Depression Scale; SCL-90, Symptom Checklist-90; PSQI, Pittsburgh Sleep Quality Index; 5-HT, 5-Hydroxytryptamine (Serotonin); IL-6, Interleukin-6; GABA, Gamma-aminobutyric Acid; DA, Dopamine. *p < 0.05, **p < 0.01.

Bold values indicate correlations significant at *p* < 0.05.

Pearson correlation coefficients were computed to examine the linear associations among study variables. To facilitate interpretation of the magnitude of observed effects, we adopted the benchmarks proposed by Gignac and Szodorai (29) for psychological research, which define correlation coefficients as small (|r| ≈ 0.10), medium (|r| ≈ 0.20), and large (|r| ≈ 0.30). These criteria are more conservative and contextually appropriate for psychological constructs than (53) conventional thresholds.

Relapse propensity showed significant positive correlations with Age (*r* = 0.322, *p* < 0.01), psychological symptoms as measured by the SCL-90 GSI (*r* = 0.261, *p* < 0.05), sleep quality problems measured by the PSQI (*r* = 0.277, *p* < 0.01), and DA concentration (*r* = 0.344, *p* < 0.01). According to Gignac and Szodorai’s (29) criteria, the correlation between relapse propensity and DA concentration represents a medium-to-large effect, while the correlations with SCL-90 GSI and PSQI represent medium effects. No other variables were significantly correlated with relapse propensity at the bivariate level.

### 3.3 Multiple linear regression analysis

To identify independent predictors of relapse propensity, a stepwise multiple linear regression was performed with all variables as potential predictors. The final model (Table 3) retained only two significant predictors: DA concentration (*β* = 0.321, *p* = 0.001) and Age (*β* = 0.301, *p* = 0.002). This model was statistically significant, *F*(2, 87) = 11.32, *p* < 0.001, and explained 18.8% of the variance in relapse propensity (Adjusted *R^2^* = 0.188). Collinearity diagnostics indicated no multicollinearity concerns (all VIFs ≈ 1.0).

**Table 3:** Stepwise multiple linear regression analysis for predictors of relapse tendency (*N* = 90).

Model	Predictor	B (Unstandardized)	SE	β	t	*p*	VIF
1	(Constant)	28.608	2.607	–	10.972	<0.001	–
	DA Concentration (nmol/l)	4.657	1.369	0.341	3.401	0.001	1
2	(Constant)	14.706	5.071	–	2.9	0.005	–
	DA Concentration (nmol/l)	4.388	1.308	0.321	3.356	0.001	1.004
	Age (years)	0.321	0.102	0.301	3.145	0.002	1.004
Model summary for each step							
Model	R	Adjusted R^2^	Δ Adjusted R^2^	F (Model)	*p* (Model)	Durbin-Watson	
1	0.341	0.106	–	11.569	0.001	–	
2	0.454	0.188	0.082	11.315	<0.001	1.507	

1. Dependent Variable: Relapse Tendency. 2. Method: Stepwise regression (Entry: *p* ≤ 0.05; Removal: *p* ≥ 0.10). 3. B: Unstandardized regression coefficient. β, Standardized coefficient. SE, Standard error. 95% CI: 95% confidence interval for B, calculated as B ± t*(0.975, df) × SE. Intervals not containing zero indicate statistical significance (*p* < 0.05), which are bolded for emphasis. 4. VIF, Variance Inflation Factor. All values are close to 1, indicating no multicollinearity concerns. 5. Δ Adjusted R^2^: The increase in explained variance from Model 1 to Model 2. 6. Excluded Variables: Other candidate variables (Education, Illness Duration, MoCA, SDS, SCL-90, PSQI, 5-HT, IL-6, GABA) did not meet the entry criterion (*p* > 0.05) in the final step (Model 2) and were not included in the final model.

The final stepwise regression model (Model 2) was statistically significant, F(2, 87) = 11.32, *p* < 0.001, and explained 18.8% of the variance in relapse propensity (Adjusted R^2^ = 0.188). The corresponding Cohen’s f^2^ effect size was calculated as f^2^ = R^2^ / (1 – R^2^) = 0.188/0.812 = 0.232, which falls within the small-to-medium range according to Cohen’s (1988) conventions (f^2^ ≥ 0.02 small, ≥ 0.15 medium, ≥ 0.35 large). The inclusion of DA concentration and age as predictors yielded a significant increment in explained variance (ΔR^2^ = 0.082, *p* = 0.002), representing a small-to-medium incremental effect.

### 3.4 Mediation analysis

**Figure 1:**
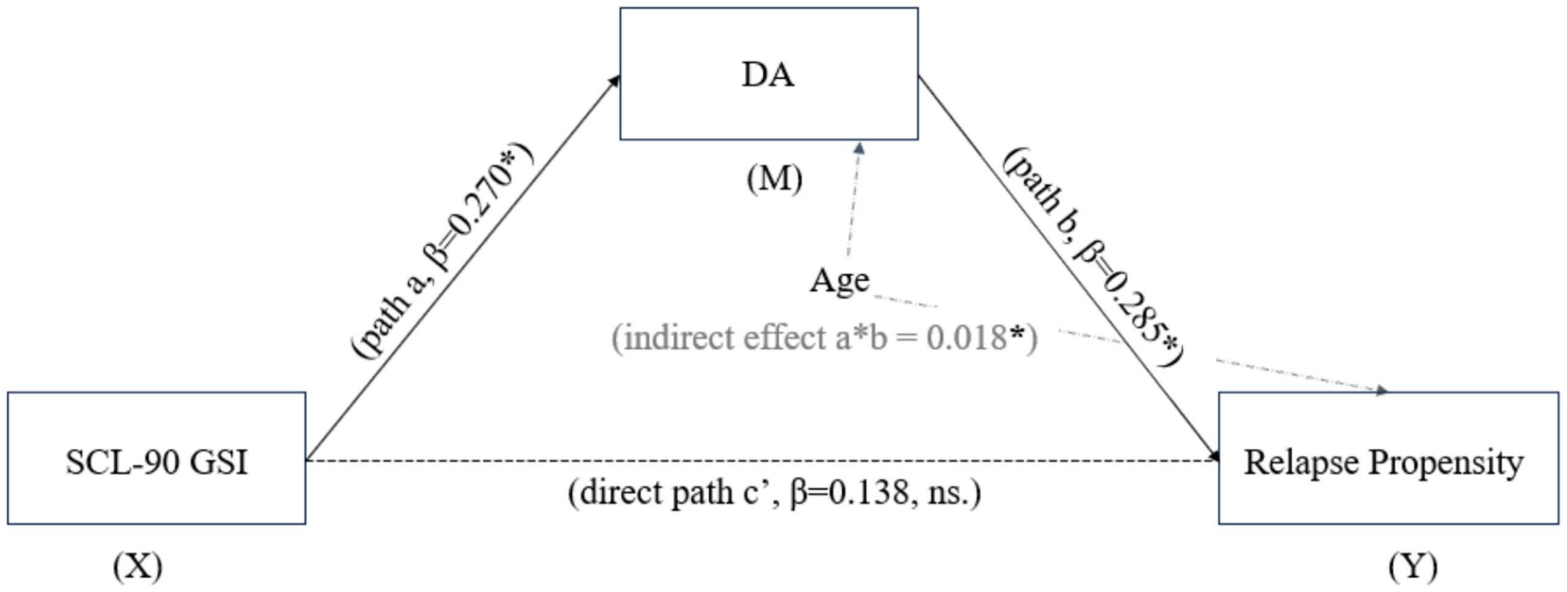
Path diagram of the mediation model. The figure presents the standard mediation triangle as the core visual: SCL-90 GSI → Dopamine (path a, *β* = 0.270*, *p* = 0.012); Dopamine → Relapse Propensity (path b, *β* = 0.285, *p* = 0.005); SCL-90 GSI → Relapse Propensity (direct path c’, *β* = 0.138, *p* = 0.170, non-significant). Age is included as a covariate, with paths pointing to both Dopamine (*β* = 0.022, *p* = 0.831) and Relapse Propensity (*β* = 0.281, *p* = 0.004). The indirect effect (a × b = 0.018, 95% CI [0.005, 0.035]) is significant, indicating full mediation. *p < 0.05, **p < 0.01.

Given the significant correlation between SCL-90 and relapse propensity and its subsequent exclusion from the final regression model in favor of DA, a mediation analysis was conducted to test whether DA concentration mediated the relationship between psychological symptoms (SCL-90 GSI) and relapse propensity, controlling for Age. The analysis used Hayes’ PROCESS macro (Model 4) with 5,000 bootstrap samples (Figure 1).

The results (Table 4) revealed a significant indirect effect. Path a was significant, indicating that higher SCL-90 GSI predicted higher DA levels (*β* = 0.270, *p* = 0.012). Path b was also significant, indicating that higher DA levels predicted greater relapse propensity (*β* = 0.285, *p* = 0.005). The bootstrap confidence interval for the indirect effect (a*b = 0.018) did not contain zero (95% CI [0.005, 0.035]), confirming a significant mediation effect. Crucially, the direct effect of SCL-90 on relapse propensity became non-significant when the mediator (DA) was included in the model (*β* = 0.138, *p* = 0.170). This pattern indicates full mediation, where the relationship between psychological symptoms and relapse propensity is entirely explained by variations in DA concentration. The mediated effect accounted for approximately 35.6% of the total effect of SCL-90 on relapse propensity.

**Table 4:** Mediation analysis testing the indirect effect of SCL-90 GSI on relapse tendency through DA concentration (controlling for age; *N* = 90).

Variable / Path	B (SE)	95% CI	β	t/z	*p*	Model fit (R^2^ / F)
Model 1 (Outcome: DA)						R^2^ = 0.075, *F* = 3.54*
SCL-90 → DA (a path)	0.005 (0.002)	[0.001, 0.008]	0.270	2.58	0.012	
Age (Covariate) → DA	0.002 (0.008)	[−0.015, 0.018]	0.022	0.21	0.831	
Constant	0.917 (0.429)	[0.064, 1.770]	–	2.14	0.036	
Model 2 (Outcome: Relapse)						R^2^ = 0.224, *F* = 8.26***
SCL-90 → Relapse (c’ path, direct)	0.033 (0.024)	[−0.014, 0.08]	0.138	1.38	0.170	
DA → Relapse (b path)	3.890 (1.350)	**[1.206, 6.573]**	0.285	2.88	0.005	
Age (Covariate) → Relapse	0.300 (0.103)	**[0.096, 0.504]**	0.281	2.92	0.004	
Constant	11.528 (5.543)	[0.510, 22.547]	–	2.08	0.041	
Total Effect (c path)	0.0508 (0.0237)	**[0.004, 0.100]**	0.215	2.15	0.035	R^2^ = 0.149, *F* = 7.60**
Indirect Effect (a × b)	0.0181 (BootSE = 0.0077)	**[0.005, 0.035]**	0.077†	–	–	

1. Model Specification: Hayes PROCESS Model 4 with 5,000 bootstrap samples for bias-corrected confidence intervals. Unstandardized. 2. regression coefficients (B) are reported with standard errors (SE) in parentheses. CI, confidence interval; β, standardized coefficient. 3. Path Labels: a path = effect of independent variable (SCL-90 GSI) on mediator (DA); b path = effect of mediator (DA) on dependent variable (Relapse Tendency) controlling for SCL-90 GSI; c’ path = direct effect of SCL-90 GSI on Relapse Tendency controlling for DA; c path = total effect of SCL-90 GSI on Relapse Tendency. 4. Significance: Bolded 95% confidence intervals (CI) do not contain zero, indicating statistical significance (*p* < 0.05). The indirect effect (a × b) is considered significant if its bootstrap CI does not contain zero. 5. Indirect Effect: †Completely standardized indirect effect (*β* = 0.077, BootSE = 0.0307, 95% BootCI [0.021, 0.142]). 6. Covariate: Age was included as a covariate in all models. *p < 0.05, **p < 0.01, ***p < 0.001.

The bootstrap confidence interval for the indirect effect (a × b = 0.018) did not contain zero (95% CI [0.005, 0.035]), confirming a significant mediation effect. The completely standardized indirect effect was *β* = 0.077 (BootSE = 0.0307, 95% BootCI [0.021, 0.142]). According to the benchmarks for mediation effects in psychological research (29, 51), this indirect effect corresponds to a small-to-medium magnitude, which is consistent with the typical effect sizes observed in complex behavioral mediation models. The mediated effect accounted for approximately 35.6% of the total effect of SCL-90 GSI on relapse propensity.

## 4 Discussion

This study employed a multi-method analytical approach to elucidate the complex interplay among psychological distress, neurobiological factors, and relapse propensity in individuals with a history of substance use disorders. The primary findings reveal a nuanced pathway: while general psychological symptom burden (SCL-90 GSI) and dopaminergic activity are both correlated with relapse risk, DA concentration emerges as the pivotal neurobiological mediator, fully accounting for the statistical association between psychological distress and relapse propensity. Age further serves as an independent contributory factor. These findings provide empirical support for an integrated “psychological-neurobiological” model of addiction relapse.

First, our correlational analysis established foundational bivariate relationships, identifying age, psychological symptoms (SCL-90 GSI), sleep quality (PSQI), and DA concentration as significant correlates of relapse propensity. This pattern aligns with extensive literature documenting the multifaceted nature of relapse vulnerability, which encompasses psychosocial, behavioral, and neurochemical domains (30, 31). Notably, the lack of significant correlation between other monoamines (e.g., 5-HT) and relapse propensity in our sample suggests a potentially more specific role for the dopaminergic system in driving motivational salience and craving in this population. This is consistent with the incentive-sensitization theory, which posits that dysregulated dopamine signaling is central to the pathological “wanting” of drugs (32, 33).

Importantly, this mediation effect must be interpreted in the context of our sample’s heterogeneity in primary substances of abuse. The sample included both stimulant users (25.6%, primarily methamphetamine) and opioid users (78.9%, primarily heroin)—two classes of substances with fundamentally different mechanisms of action on the dopaminergic system. Stimulants (e.g., methamphetamine) directly increase synaptic dopamine by blocking dopamine reuptake via the dopamine transporter and promoting reverse efflux from vesicular stores (34). In contrast, opioids (e.g., heroin) indirectly modulate dopamine neurons by activating *μ*-opioid receptors on GABAergic interneurons in the ventral tegmental area, thereby disinhibiting dopamine neurons and increasing dopamine release in the nucleus accumbens (35).

Despite these mechanistic differences at the molecular level, both pathways converge on a common final outcome: elevated dopamine signaling in mesolimbic circuits. The fact that the mediation effect holds across this heterogeneous sample suggests that psychological distress may act through a trans-diagnostic mechanism—dopamine dysregulation—that represents a final common pathway to relapse vulnerability, regardless of the specific pharmacological actions of the primary substance of abuse. This interpretation is consistent with the incentive-sensitization theory, which posits that sensitized dopamine responses to drug cues, rather than the direct hedonic effects of drugs, underlie pathological “wanting” and compulsive drug-seeking behavior (32). From this perspective, psychological distress may serve as an endogenous trigger that sensitizes the dopamine system, amplifying cue-induced craving and relapse risk across different substance use disorders.

The stepwise regression results provided critical refinement. When all variables were considered simultaneously, only DA concentration and age retained significant independent predictive value for relapse propensity. The exclusion of SCL-90 and PSQI from the final model suggests that the predictive power of these psychological constructs may not be direct but is largely shared with, or conveyed through, their association with the neurobiological substrate—DA. This statistical observation formed the empirical premise for testing a formal mediation model (36).

The central and most significant finding is the demonstration of a full mediation effect. Our analysis conclusively showed that the relationship between general psychological symptom burden and increased relapse propensity is entirely mediated by peripheral DA concentration. This result provides a plausible neurobiological mechanism for the well-established clinical observation that comorbid psychological distress exacerbates addiction outcomes (37, 38). We posit that psychological symptoms (e.g., anxiety, depression, hostility) may act as a chronic stressor. In line with the “stress-allostasis” model of addiction (3), such distress could induce neuroadaptations within the mesolimbic dopamine system and associated circuits, leading to a hyper-reactive dopaminergic state that favors conditioned responses to drug cues (8, 15). This sensitized or dysregulated state, in turn, is theorized to amplify the incentive salience of drug-related cues and increase the motivational drive to use, thereby elevating relapse propensity. Our finding that the direct path from symptoms to relapse became non-significant after accounting for DA underscores dopamine’s potential role as a final common pathway in this process, integrating affective distress with motivated behavior (39).

The independent contribution of age to relapse propensity, alongside DA, warrants attention. This may reflect cumulative neurobiological adaptations (e.g., reduced striatal D2/D3 receptor availability) and life-course changes (e.g., diminished cognitive control, entrenched habits) associated with prolonged substance use and aging itself, factors not fully captured by DA concentration alone (40, 41). The mean age of our sample (44.73 years) and the predominance of long-term drug use (>10 years for 70% of participants) are consistent with the demographic profile of compulsory rehabilitation populations in China, where drug use trajectories often involve decades of intermittent use and multiple treatment episodes (42, 43).

Our findings should also be considered in the context of existing research from similar socio-cultural settings. Studies conducted in Chinese compulsory rehabilitation facilities have consistently reported elevated rates of psychological distress, with SCL-90 scores in our sample (raw mean item score = 1.69, calculated as total score/90) comparable to those reported in other Chinese studies of women with substance use disorders (44). This raw mean score ranges from 0 to 4, with higher scores indicating greater psychological distress, and is the standard metric used in research comparisons, distinct from T-scores typically used for clinical diagnostic purposes. Our study extends this literature by demonstrating that this relationship is not direct but operates through dopamine dysregulation—a neurobiological mechanism that has rarely been examined in non-Western samples. This convergence with findings from Western populations (31) suggests that the psychological-neurobiological pathway to relapse may transcend cultural boundaries, although future cross-cultural comparative studies are needed to test this hypothesis directly.

### 4.1 Strengths

A key strength of this study is the integration of validated psychological measures with quantitative neurochemical assays within a formal mediation framework, moving beyond correlation to propose a testable mechanistic pathway. The use of bias-corrected bootstrap confidence intervals enhances the robustness of our mediation finding (45). Key strengths include: (1) integration of psychological measures with neurochemical assays within a formal mediation framework; (2) use of bootstrap confidence intervals for robust mediation testing; and (3) focus on an understudied population—women in compulsory drug rehabilitation.

### 4.2 Limitations

Several limitations should be considered when interpreting these findings. First, the cross-sectional design precludes causal inference. Longitudinal studies are needed to establish temporal precedence. Second, peripheral dopamine measurement may not reflect central dopaminergic function. Future research should employ neuroimaging (e.g., PET) to directly assess brain dopamine activity. Third, the all-female sample limits generalizability to males. Given sex differences in dopamine function, comparative studies including both sexes are warranted. Fourth, the modest sample size (*N* = 90) limited statistical power to detect small effects and precluded stratified analyses by substance type (stimulants vs. opioids). Larger samples are needed for subgroup analyses. Fifth, other limitations include single-center recruitment and use of a self-report relapse scale rather than objective relapse events.

### 4.3 Future directions

Building on these findings and addressing the limitations outlined above, future research should: (1) use longitudinal designs to establish causality; (2) employ neuroimaging to assess central dopamine function; (3) recruit larger, diverse samples to enable subgroup analyses by substance type and gender; and (4) conduct intervention studies to test whether targeting psychological symptoms or dopamine modulation reduces relapse risk.

### 4.4 Clinical implications

These findings have direct translational implications. They argue against a siloed approach to treating addiction and co-occurring psychological distress. Instead, they support integrated, dual-target treatment strategies that concurrently address psychological symptoms and their potential downstream neurobiological consequences (46). For instance, psychotherapies effective in reducing general distress and improving emotion regulation (e.g., Dialectical Behavior Therapy, Acceptance and Commitment Therapy) may exert part of their beneficial effect on relapse by helping to normalize stress-induced dopaminergic hyperactivity (47, 48). Conversely, interventions directly or indirectly aimed at stabilizing dopaminergic signaling (e.g., certain pharmacotherapies, neuromodulation approaches like repetitive Transcranial Magnetic Stimulation) could indirectly ameliorate the relapse risk associated with psychological symptoms (49, 50). Assessment of relapse risk may be refined by considering both psychological state and potential neurobiological vulnerability markers.

## 5 Conclusion

This study elucidates a critical neurobiological pathway linking psychological distress to substance relapse vulnerability. Through integrated correlational, regression, and mediation analyses, we demonstrate that dopamine concentration serves as a full and specific mediator of the relationship between general psychological symptom burden and heightened relapse propensity, while age contributes additional independent predictive power. This finding reframes psychological symptoms not merely as comorbid conditions but as key drivers of neurochemical dysregulation that directly fuels relapse risk. It establishes the dopaminergic system as a pivotal convergence point where psychosocial adversity translates into neurobiological vulnerability.

These results have significant theoretical and clinical implications. Theoretically, they provide a mechanistic neurochemical framework for the established clinical link between psychological comorbidity and poor addiction outcomes. Clinically, they argue compellingly for integrated, biopsychosocial intervention models that concurrently target psychological distress and its downstream neurobiological consequences. Future research should prioritize longitudinal and interventional designs to establish causality, examine the specificity of symptom domains, and explore the utility of peripheral DA as a potential biomarker for relapse risk stratification. Ultimately, this work advances a more precise, mechanism-informed understanding of addiction relapse, moving the field toward personalized treatment strategies that address the unique psychological and neurobiological profile of each individual.

## Data Availability

The raw data supporting the conclusions of this article will be made available by the authors, without undue reservation.
